# Magnetic Microrobots with Folate Targeting for Drug Delivery

**DOI:** 10.34133/cbsystems.0019

**Published:** 2023-05-05

**Authors:** Min Ye, Yan Zhou, Hongyu Zhao, Xiaopu Wang

**Affiliations:** Shenzhen Institute of Artificial Intelligence and Robotics for Society (AIRS), The Chinese University of Hong Kong, Shenzhen, Guangdong 518129, China.

## Abstract

Untethered microrobots can be used for cargo delivery (e.g., drug molecules, stem cells, and genes) targeting designated areas. However, it is not enough to just reach the lesion site, as some drugs can only play the best therapeutic effect within the cells. To this end, folic acid (FA) was introduced into microrobots in this work as a key to mediate endocytosis of drugs into cells. The microrobots here were fabricated with biodegradable gelatin methacryloyl (GelMA) and modified with magnetic metal–organic framework (MOF). The porous structure of MOF and the hydrogel network of polymerized GelMA were used for the loading of enough FA and anticancer drug doxorubicin (DOX) respectively. Utilizing the magnetic property of magnetic MOF, these microrobots can gather around the lesion site with the navigation of magnetic fields. The combination effects of FA targeting and magnetic navigation substantially improve the anticancer efficiency of these microrobots. The result shows that the cancer cells inhibition rate of microrobots with FA can be up to 93%, while that of the ones without FA was only 78%. The introduction of FA is a useful method to improve the drug transportation ability of microrobots, providing a meaningful reference for further research.

## Introduction

At present, untethered microrobots have achieved remarkable achievements in minimally invasive surgery [1,2], drug delivery [3–5], environmental remediation [6–8], and tissue engineering [9,10]. These microrobots can be actuated wirelessly by external energy such as magnetic field [3,11,12], light [13–15], ultrasound [16,17], chemical reaction [18–20], electric field [21], etc. Among the numerous driving energy sources, the magnetic field has good biosafety, deeper tissue penetration, and a large control range. By controlling the direction and intensity of the magnetic field, the magnitude and direction of the torque applied on the microrobot would be adjusted to guide the microrobot's motion [22]. This drive mode presents a great ability with high temporal and spatial control and fast response.

For the sake of safety for biomedical applications, the development of biodegradable magnetic microrobots can avoid the practical problem that microrobots are difficult to recycle and may cause side effects to the human body. To this end, many biodegradable microrobot systems have been developed [3,23–25]. Whereinto, the newly developed one based on gelatin methacryloyl (GelMA) and iron-doped zeolitic imidazolate framework (Fe@ZIF-8) hold great potential for cargo delivery [26]. Due to the 3-dimensional (3D) network structure of GelMA hydrogel and the high-porosity structure of Fe@ZIF-8, this system has a strong cargo loading capacity [27–30]. Moreover, the good magnetic properties of metal–organic framework (MOF) also provide the prerequisite for the magnetic actuation of microrobots. Magnetic artificial bacterial flagellar (ABF) microrobots, which have the design of microhelical structures, can convert their nonreciprocal rotating motion into a translational motion under a rotating magnetic field, so as to push themselves forward efficiently [31–33]. However, after reaching the designated position, microrobots generally cannot assist drugs to enter cells. With only the manner of free diffusion, the speed and concentration of drugs entering cells are limited, leading to a relatively poor therapeutic efficacy.

To endow the microrobot with specific cancer cell targeting ability and to promote the ingestion of drugs by cells, a commonly used cancer-targeting molecule folic acid (FA) was introduced to microrobots in this work. FA is a small-molecule ligand with high bioaffinity, which could specifically recognize folate receptor (FR) [34–36]. The selective overexpression of the FR on the surface of cancer cells provides FA the ability to promote the uptake of drugs by cancer cells through receptor-ligand-mediated endocytosis [37–39]. By combining FA with the microrobot, the resulting drug delivery system can not only locate the lesion area with the control of magnetic fields but also bring the loaded drug into the cytoplasm through endocytosis.

**Fig. 1.**
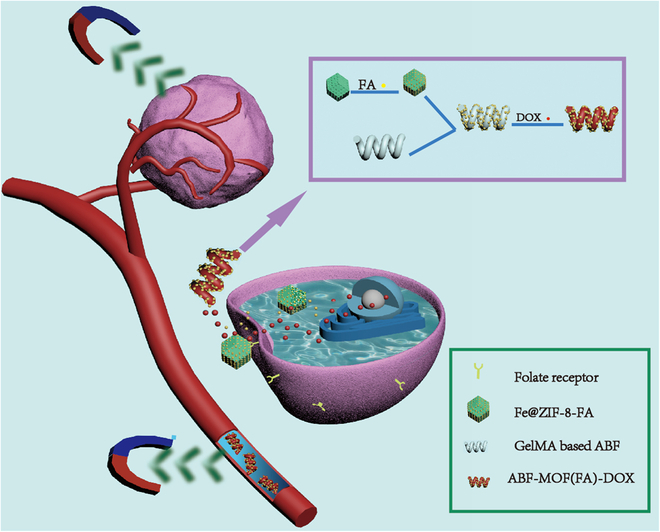
The illustration of magnetically controlled ABF microrobots for folate-targeted cancer therapy.

In this work, a folate-targeting magnetic microrobot system that consists of biodegradable GelMA-based ABF microhelix and FA-loaded Fe@ZIF-8 nanoparticles was developed, for which therapeutic drugs can be loaded into the hydrogel network of the microrobots for cancer therapy (Fig. 1). For simplification, Fe@ZIF-8 is generally marked as MOF in this work, unless otherwise specified. Thus, the developed system can be represented by ABF-MOF(FA), and ABF-MOF(FA)-DOX (doxorubicin) represents this system loaded with therapeutic drugs (DOX). With the directional manipulation of an external magnetic field, the microrobot can be navigated and fixed at the lesion site to ensure therapeutic drugs gather around cells. The full binding of FA on the microrobots and FR on the surface of cancer cells can trigger the occurrence of endocytosis, resulting in the entering of MOF(FA) and DOX into the cells. Accumulation of microrobots near cells is beneficial to the interaction of receptor and ligand, which can further improve the therapeutic efficiency. The results indicated that microrobots with FA showed more obvious cell inhibition compared to those without FA. Therefore, the ABF-MOF(FA) drug delivery system, which combines magnetic manipulation and the active targeting of FA, has a promising application prospect for cancer treatment.

## Materials and Methods

### 3D printing of the helix microstructures

GelMA, a derivative of type B gelatin, not only has biodegradability but also can be photopolymerized. Combined with 2-photon lithography technology, submicron GelMA spiral microstructures can be prepared for drug delivery [40,41]. The GelMA-based microstructures in this work were fabricated using direct-laser-writing system Nanoscribe GT2, following the process in previous literatures [40]. Specifically, GelMA (200 mg/ml) and sodium photoinitiator 3,3′-(((1E, 1′E)-(2-oxcyclopentane-1,3-diylidene) bis (methanylylidene)) bis (4,1-phenylene) bis (methylazanediyl)) dipropanoate (P2CK) (1 mg/ml) were dissolved in phosphate-buffered saline (PBS) buffer at 45 °C to prepare the precursor solution (all the PBS mentioned in this work is 1×PBS). A drop of the precursor solution was dropped onto the center of a glass substrate, and then the direct laser writing was performed using a 25× objective lens with a laser power of 50 mW and a scan speed of 30,000 μm/s, setting a piezo settling time of 10 s and a galvo acceleration of 1 v/ms^2^. During the printing, a near-infrared femtosecond laser was used as the light source to scan in the precursor solution droplet to induce the photopolymerization. The scanning trajectory of the near-infrared laser is determined by the code generated by Describe (a professional software provided by the Nanoscribe company) according to the 3D model of the designed microrobot. The microrobot was designed to have the body length of 140 μm and the screw diameter of 50 μm. After the printing, the sample was placed in PBS at 45 °C for 30 min to remove the unpolymerized precursor solution to obtain the micro helixes.

### The synthesis of MOF and MOF(FA)

The Fe@ZIF-8 (MOF) was synthesized according to the protocol reported by Terzopoulou et al. [26]. FeSO_4_.7H_2_O (2.5 ml; 0.43 M) and 2.5 ml of Zn(OAc)_2_.2H_2_O (0.55 M) were mixed and then added to 5 ml of 2-methylimidazole (5.5 M) aqueous solutions (all solutions were prepared with deionized water). Under sealing conditions, the mixture solution was stirred for 24 h at room temperature. Then, the reaction solution was subsequently washed several times with deionized water and lyophilized to obtain the final product. Fe@ZIF-8 (FA) (MOF(FA)) was prepared with the following protocol: 20 mg of FA was dissolved in 20 ml of dimethyl sulfoxide, followed by adding 20 mg of MOF. The mixture was treated by sonication for 1 h, followed by stirring at 500 rmp for 24 h. Excess-free FA was removed by repeated centrifugation washing steps. Finally, a black solid product was obtained after freeze-drying with a lyophilizer (scientz-12N/A). The FA loading rate was calculated as follows:

$$\text{Loading rate}\left(\%\right)=\frac{\mathrm{MOF}{\left(\mathrm{FA}\right)}_{\text{total}}-{\mathrm{MOF}}_{\mathrm{add}}}{\mathrm{MOF}{\left(\mathrm{FA}\right)}_{\text{total}}}\times 100\%$$

### Assembly of the ABF microrobot

The microrobots were assembled as follows: the above-printed ABF microstructures were soaked in a MOF(FA) or MOF solution at a concentration of 20 mg/ml for 3 h to obtain microrobots fully decorated with magnetic nanoparticles (ABF-MOF(FA)/ABF-MOF). Excess MOF(FA) or MOF was removed by washing with PBS buffer more than 3 times. The magnetic microrobots were then immersed in 2 mg/ml DOX aqueous solution at 45 °C for 2.5 h to obtain a microrobot (ABF-MOF(FA)-DOX or ABF-MOF-DOX) loaded with the therapeutic drug DOX. Excess DOX was washed away with PBS buffer (4 °C) several times. PBS buffer with low temperature was used here to avoid the release of DOX, which has been loaded in the microrobots, in the washing step.

### Characterization

After preparation and assembly steps, optical microscope images of GelMA-based ABF-MOF(FA) and ABF-MOF(FA)-DOX were taken for feature validation. Furthermore, we confirmed the successful preparation of MOF by a scanning electron microscope (SEM). Physical property test was done to characterize the magnetism of MOF and MOF(FA) using a physical property measurement system (PPMS-VSM). Subsequently, to prove the successful loading of FA in MOF, fluorescence luminescences of MOF(FA), MOF, and FA aqueous solutions at 365-nm excitation were measured and compared with each other, with the equipment of a Synergy H1 multimode microplate reader (BioTek Instruments).

### Drug loading and releasing properties

The drug release capacity of ABF-DOX was characterized. Initially, we soaked 500 ABFs in 2 mg/ml DOX aqueous solution at 45 °C for 2.5 h and then washed them with PBS buffer solution several times to obtain the preliminary drug-loading system. Then, we immersed them in PBS with pH = 5.3 and conducted a drug release test at 37 °C. At selected time intervals, 500 μl of release solution was collected for testing and supplemented with an equal volume of fresh PBS buffer solution. The amount of the released DOX was evaluated by measuring the absorption of the release solution at 479 nm with ultraviolet (UV)/visible spectrophotometry. All experiments were repeated 3 times independently to minimize bias.

### In vitro cytotoxicity (MTT)

To evaluate the targeting effect of FA in the anticancer process, the MTT (3-(4,5-dimethylthiazol-2-yl)-2,5-diphenyltetrazolium bromide) method was used to detect the cytotoxicity of therapeutic drugs with human breast cancer (MCF-7) cells. MCF-7 cells were seeded into a 48-well plate (15,000 cells per well) and cultured in a cell culture medium (90% Dulbecco's modified eagle medium, 10% fetal bovine serum, and 0.5% cyanine streptomycin antibacterial) under physiological conditions (37 °C and 5% CO_2_) for 24 h. Then, a mixture of MOF(FA) (0.05 mg/ml) and DOX (10 μg/ml or 5 μg/ml) was added to the cell cultures to be the experimental groups. The control group was distinguished by replacing the MOF(FA) with the MOF. The cell viability percentage was calculated by MTT viability assay at 24 and 48 h after the treatment. For the MTT assay, the experiment samples were treated with 5 mg/ml MTT solution prepared with PBS buffer solution and incubated at 37 °C for 4 h. Then, dimethyl sulfoxide (100 μl per well) was added to dissolve the formazan deposited in the living cells. The light absorption values of the samples were measured at 490 nm with a full-wavelength multifunctional microplate detector (Synergy H1) to indirectly reflect the number of living cells.

### Anticancer efficiency of microrobots

We evaluated the inhibitory effect of the microrobot drug delivery system on MCF-7 cells through live/dead cell staining experiments, in which the living cells appeared in green and the dead cells appeared in red in the fluorescence images. The MCF-7 cells and microrobots were seeded into a 6-well plate. After incubation of 24 and 48 h, the culture medium was replaced by 2 μM calcein-AM and 4 μM propidium iodide. Then, the samples were incubated for 30 min, followed by being washed with PBS buffer solution. Finally, the fluorescence images were recorded using an inverted fluorescence microscope (Olympus x75).

### Magnetic actuation and control of the microrobots

The magnetic field generator (MFG-100, Magnebotix, Zurich, Switzerland) was used to generate a rotating magnetic field for the movability test of the microrobots. A microprobe (T-4-22 model) was used to release and place microrobots on a clean petri dish subtract. Within a rotating magnetic field of 20 mT, the moving performance of the microrobots was observed and evaluated. The movability test of microrobots was also demonstrated in both a cross-shaped microchannel and a petri dish with cells landed inside.

### Fabrication of microchannel

**Fig. 2.**
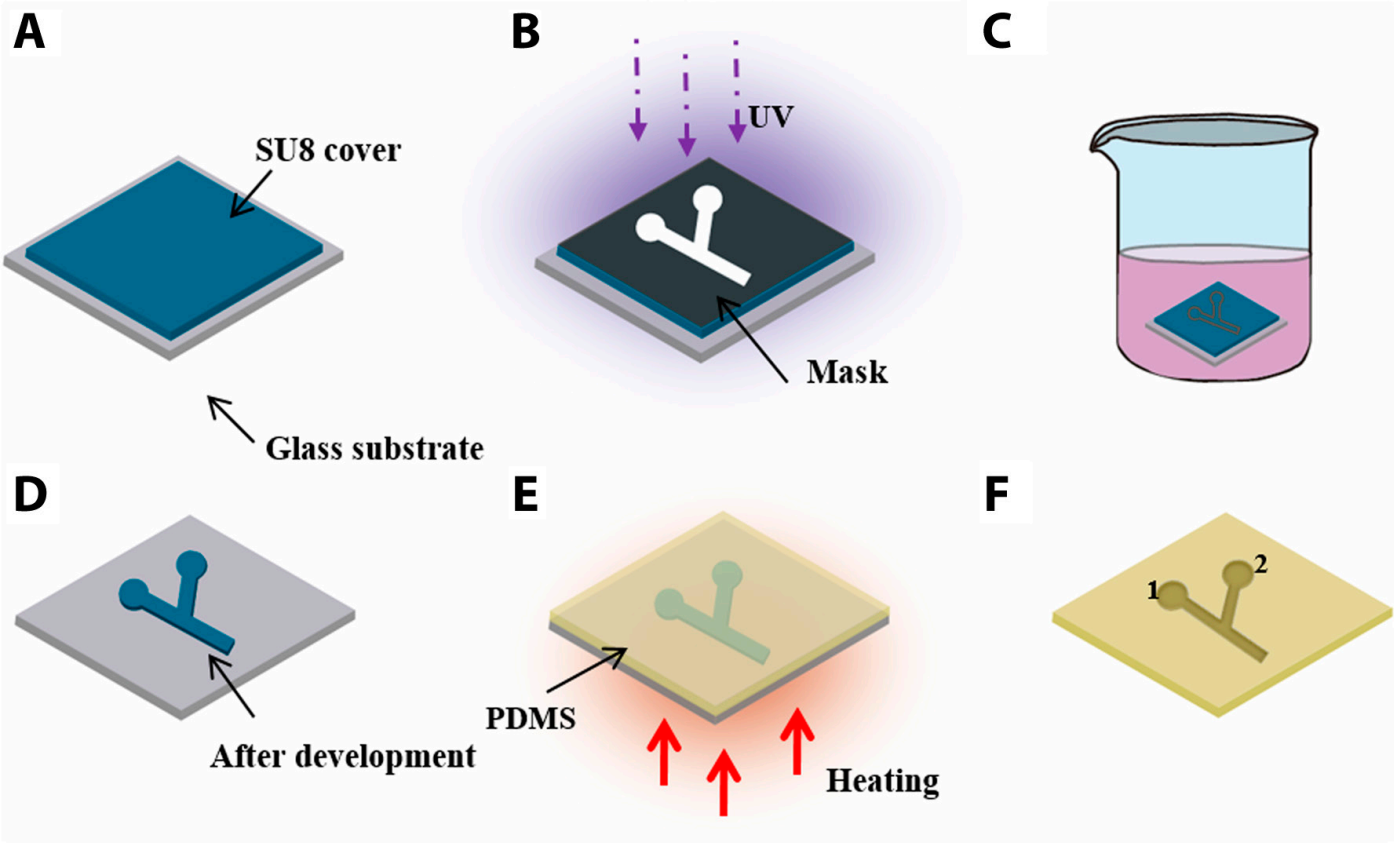
Schematic illustration of the Y-shape microchannel fabrication process. (A) A layer of SU-8 coated on a glass substrate. (B) UV polymerization of SU-8 with a Y-shape photomask. (C) Development of photopolymerized SU-8. (D) The obtained SU-8 Y-shape pattern. (E) Heating of PDMS to fabricate the microchannel. (F) The obtained Y-shape PDMS microchannel.

As shown in Fig. 2, a Y-shaped microchannel was fabricated to be used for an application demonstration, within which drug-loaded microrobots were navigated by a magnetic field to a target area to exert the anticancer effect. First, a layer of SU-8 2150 photoresist (500 μm) was coated on the glass substrate with a spin coater (Fig. 2A). Then, the photoresist was polymerized by UV irradiation for 30 s with a photomask that has a Y-shape pattern (Fig. 2B). Next, after removing the photomask, the sample was developed within a solution of propylene glycol methyl ether acetate (Fig. 2C). As the result, a clear Y-shape SU-8 pattern attached to the glass substrate was obtained (Fig. 2D). Afterward, the glass substrate was covered by a layer of polydimethylsiloxane (PDMS) solution and heated at 110 °C for 1 h (Fig. 2E). In the end, a PDMS Y-shaped microchannel was obtained by removing the glass substrate with the SU-8 pattern. The areas at both ends of the microchannel are marked as hole 1 and hole 2, respectively (Fig. 2F).

### Application demonstration experiment

Within the Y-shaped microchannel, 1,000 MCF-7 cells were seeded in hole 1 and hole 2. After 24 h, the cells adhered to the wall and grew normally. Then, 10 microrobots were navigated toward hole 1 with a rotating magnetic field (20 mT, 6 Hz), while no microrobots were guided toward hole 2. After the microrobots reached hole 1, the cells in the microchannel were continuously incubated for another 24 h. After the entire 48-h incubation, dead cells were stained with propidium iodide, and the survival states of the cancer cells were observed with a fluorescence microscope.

## Results and Disscussion

### Fabrication and characterization

**Fig. 3.**
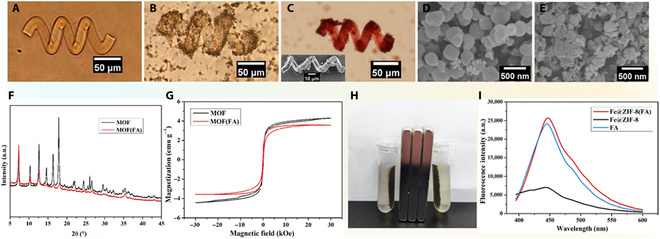
(A and B) The optical images of ABF and ABF-MOF(FA), respectively. (C) The optical image and SEM image of ABF-MOF(FA)-DOX. (D and E) The SEM image of MOF and MOF(FA). (F) XRD analysis of MOF and MOF(FA), respectively. (G) Magnetization curves of soft magnetic MOF and MOF(FA). (H) Collection of MOF (left) and MOF(FA) (right) with a magnet. (I) Fluorescence emission spectra of MOF(FA), MOF, and pure FA.

Figure 3A to C shows the optical images of a 3D printed microhelix, an assembled magnetic microrobot, and a drug (DOX)-loaded microrobot, respectively. The main body of the microrobot is transparent (Fig. 3A). With the uniform distribution of MOF(FA) on the surface of the microrobot, its optical image becomes darker (Fig. 3B). After DOX loading, the color of the microrobot framework turns to be red, indicating that DOX was loaded successfully into the hydrogel network of the microrobot (Fig. 3C), and the inset in Fig. 3C is a SEM image of ABF-MOF(FA)-DOX. It can also be noted that the micro helix becomes slightly smaller after DOX loading. This might mainly be because of the shrinkage of GelMA caused by dehydration during multiple cleaning steps with PBS (4 °C). Figure 3D shows the SEM image of MOF, which has a typical ZIF-8 dodecahedron shape with particle size values of 300 ± 50 nm. After FA loading, the structure of MOF(FA) becomes irregular (Fig. 3E), and the crystallinity is reduced, as shown in the x-ray diffraction (XRD) results (Fig. 3F). However, the VSM result (Fig. 3G) indicates that the ferrimagnetism of these nanoparticles does not change obviously because of folate loading. Using the calculation method mentioned above, the loading rate of FA in MOF is about 10.2%. When dispersed in water, MOF and MOF(FA) can both be quickly collected with magnets, which ensures the effective separation and collection of them (Fig. 3H). In addition, to verify that FA has been successfully loaded in MOF, the emission spectra of MOF-FA, MOF, and FA were investigated. As shown in Fig. 3I, being consistent with pure FA, MOF(FA) has the strongest emission peak at 450 nm under the excitation wavelength of 365 nm, while MOF without FA has no such fluorescence characteristics. Based on the above results, the microrobot has 2 substantial advantages: (a) the whole system is very suitable as a cargo carrier and its assembly process is easy; and (b) compared with other metal coating used solely to provide magnetism for microrobots, magnetic MOF can provide both magnetism and cargo loading ability for microrobots.

### Drug loading and release properties

**Fig. 4.**
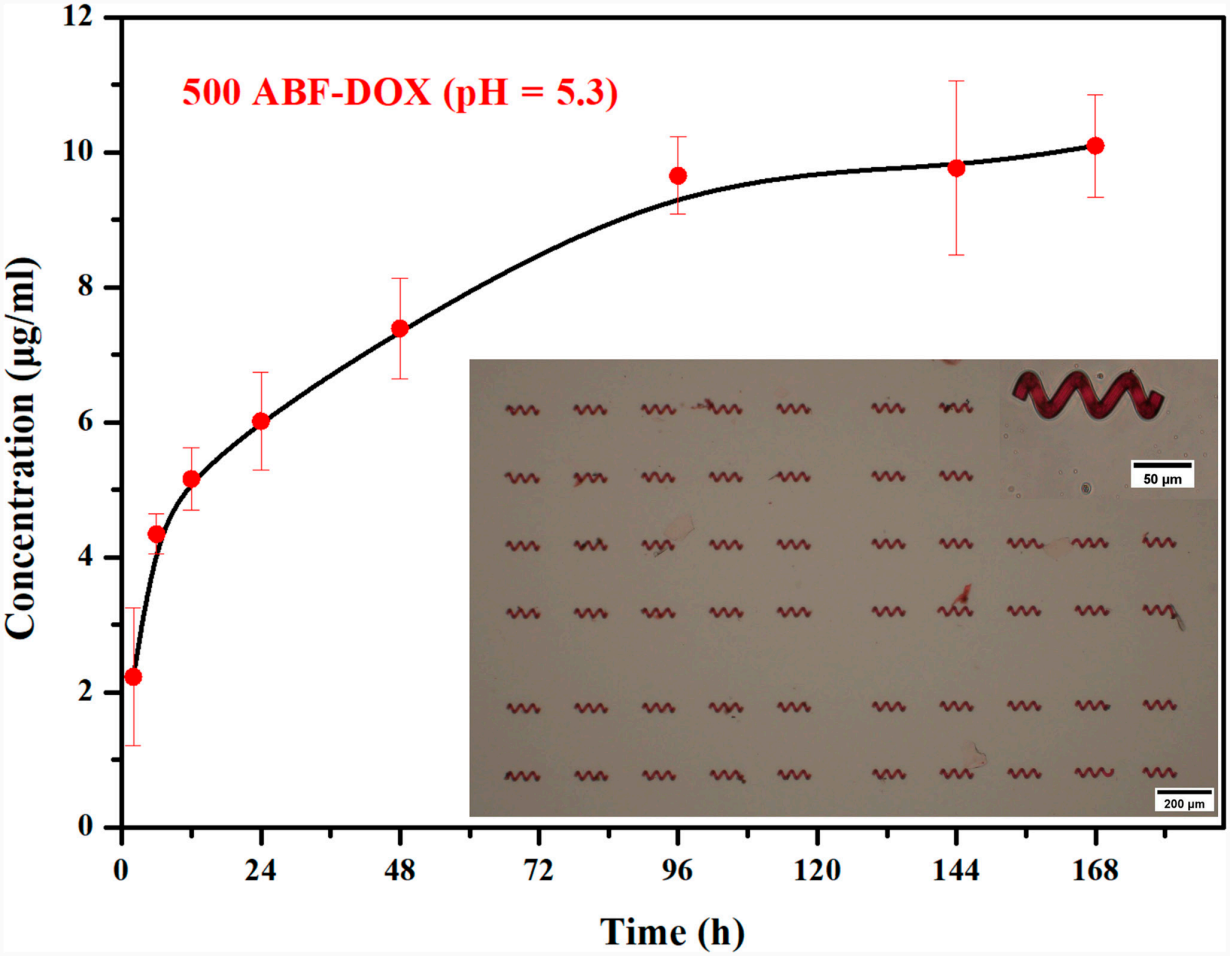
Drug release curve of ABF-DOX at 37 °C and pH 5.3.

As the surrounding environment of cancer cells is weakly acidic, the drug release ability of GelMA-based microstructures was tested at acidic environment here. Figure 4 illustrates the curve of DOX release from GelMA-based microstructures in PBS buffer with pH = 5.3. At a constant temperature of 37 °C, the release amount of DOX gradually increased in the first 96 h and then began to grow slowly. This result indicates that the microrobot based on GelMA has a certain drug encapsulation ability and drug release ability.

### In vitro cytotoxicity (MTT)

**Fig. 5.**
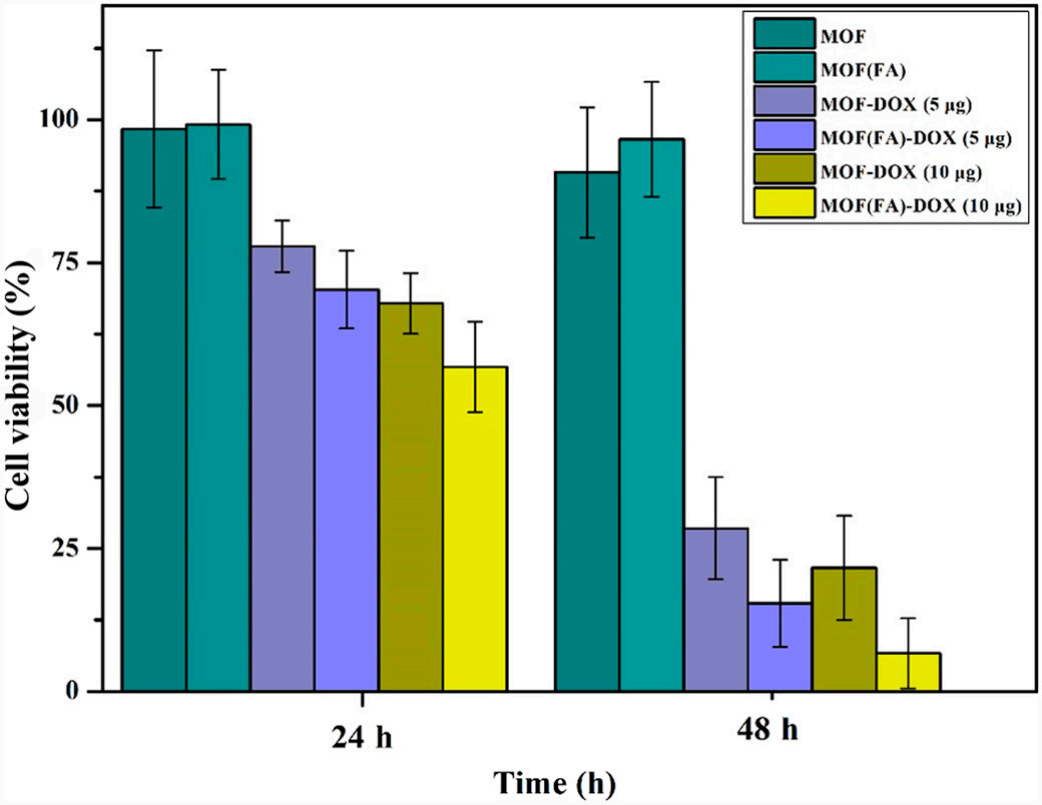
Viability analysis of MCF-7 cells treated with different samples for 24 and 48 h.

According to the drug release data in Fig. 4, a relatively large concentration (10 μg/ml) and a relatively intermediate concentration (5 μg/ml) of DOX that can be released by the microhelix were selected in the test groups here and the concentrations of MOF and MOF(FA) were selected as 0.05 mg/ml. As depicted in Fig. 5, high cell viability (close to 100%) of MOF and MOF(FA) groups was observed, indicating the biocompatibility of MOF and FA. In contrast, relatively low cell viability of MOF-DOX groups and MOF(FA)-DOX groups indicates their substantial growth inhibition to cancer cells, and high DOX concentration also results in better inhibition. Under the same experimental conditions and culture time, the survival rate of cancer cells in the group with FA was distinctly lower than that without FA, which was attributed to endocytosis of DOX mediated by the binding of FA to FR overexpressed on the surface of cancer cells. With more anticancer drugs entering the cell and acting on the nucleus, groups with FA have better cancer cell inhibitory effect. Taking the cases with 10 μg/ml DOX and 48-h treatment, the MOF(FA)-DOX group shows a marked cell growth inhibition with 7% cell survival rate, while the cell survival rate of the MOF-DOX group is 22%. The MTT results verify that FA in our microrobot system plays an important role in enhancing the targeting therapeutic effect.

### Anticancer efficiency of microrobots

**Fig. 6.**
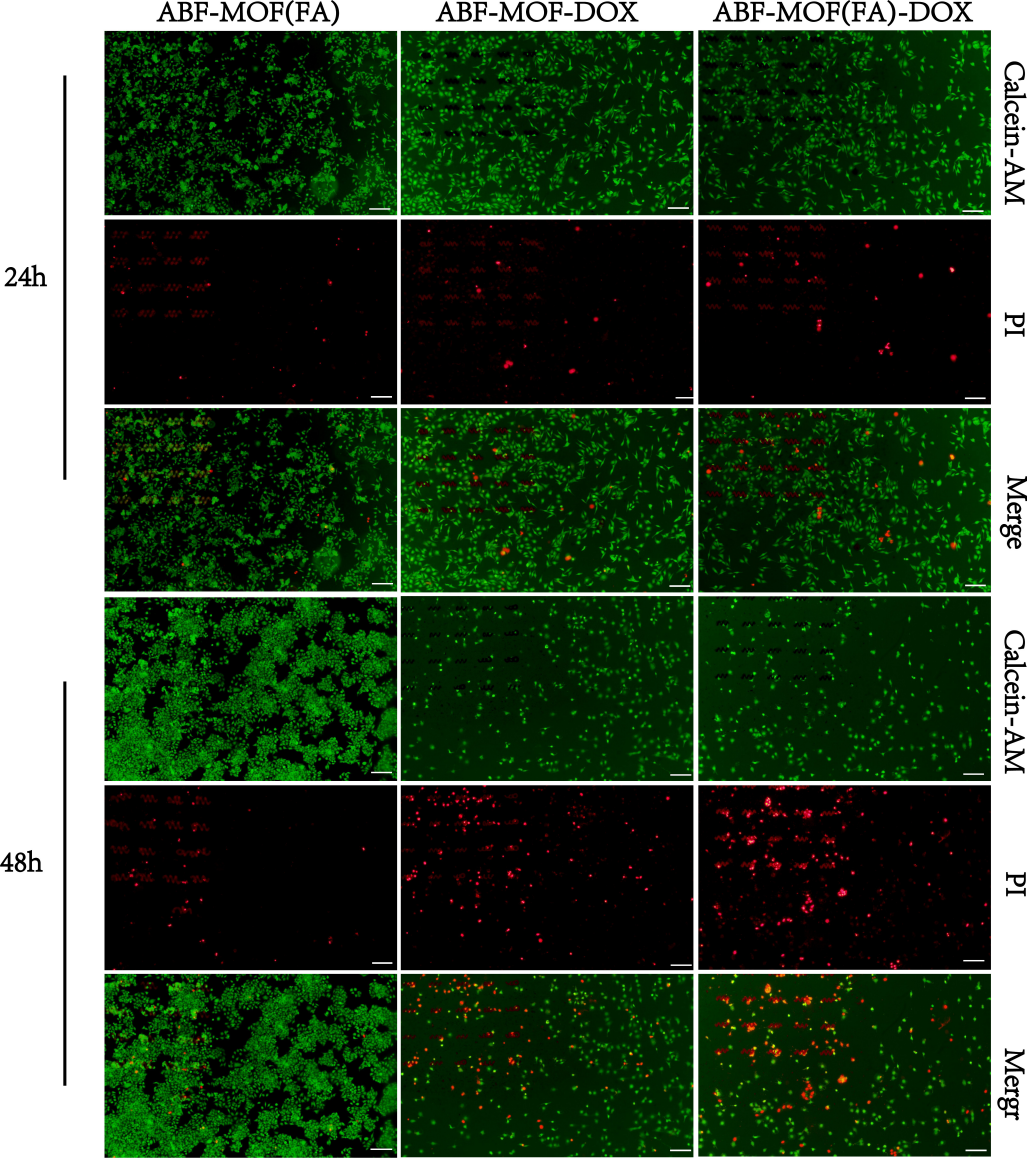
Live/dead staining images of MCF-7 cells treated with ABF-MOF(FA), ABF-MOF-DOX, and ABF-MOF(FA)-DOX for 24 and 48 h. Scale bar, 200 μm. PI, propidium iodide.

To demonstrate the inhibitory effect of drug-loaded microrobots on breast cancer cells, live/dead cell viability assays have been used for the evaluation. As illustrated in Fig. 6, MCF-7 cells incubated with ABF-MOF(FA) (the control group) grew normally, and notable proliferation was observed within 48 h. This suggested that the microrobots based on GelMA, MOF, and FA had good biocompatibility for MCF-7 cells and barely affect cell growth. Contrasting to the continuously increasing live cell density of the control group, the live cell densities of the ABF-MOF(FA)-DOX and ABF-MOF-DOX groups were both decreasing over time. This indicated that the therapeutic drug DOX can be effectively released from the microrobot and plays a role in inhibiting MCF-7 cells. Furthermore, the live cell density in the ABF-MOF(FA)-DOX group is less than that in the ABF-MOF-DOX group, while the dead cell densities in these 2 groups show an opposite relationship, especially at 48 h. This result is consistent with the previous assumption that the introducing of FA improves the tumor targeting ability of the microrobot system, enhancing the therapeutic effect.

### Magnetic actuation and control of the microrobots

**Fig. 7.**
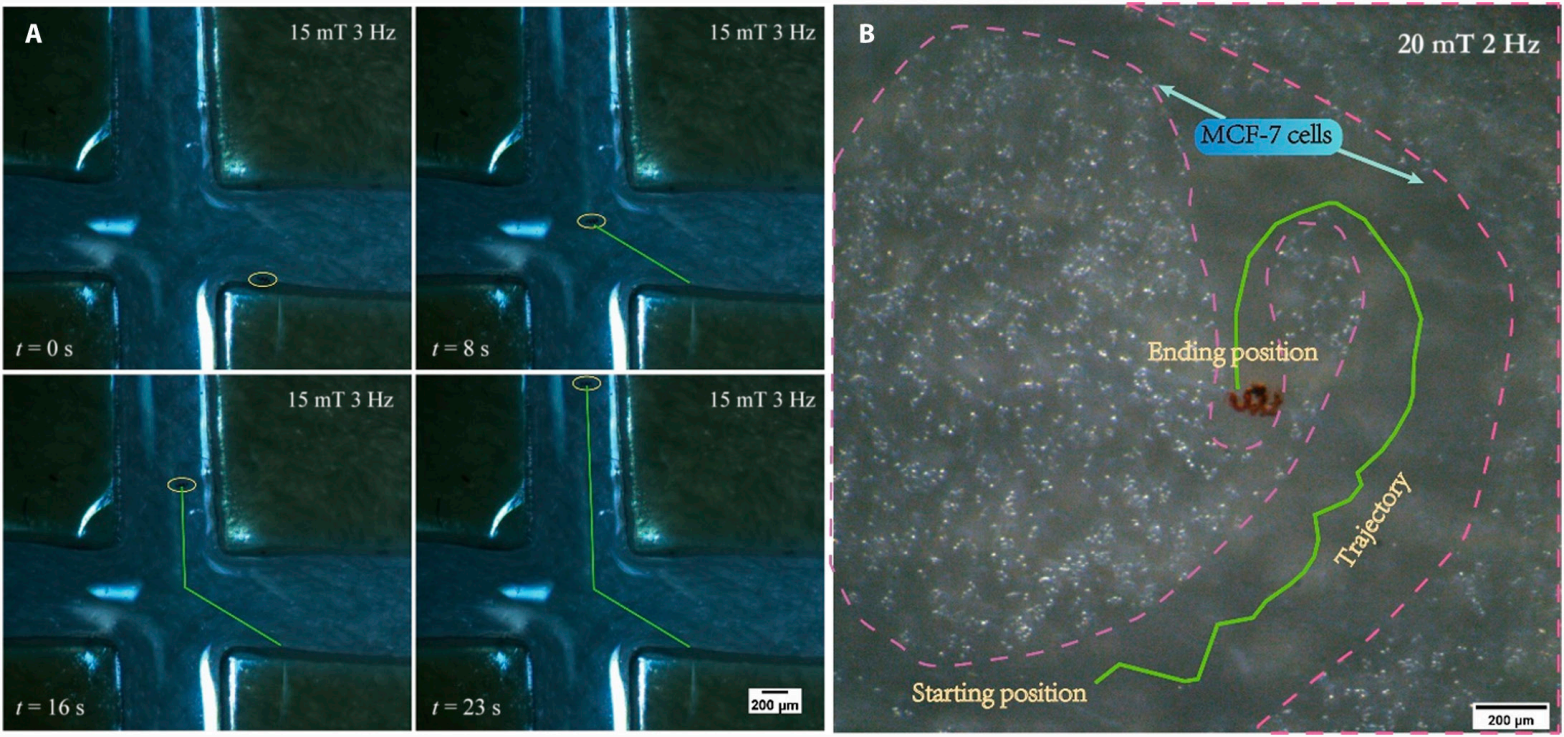
(A and B) The swimming trajectory images of a ABF-MOF(FA)-DOX microrobot was driven by a rotating magnetic field in the microchannel and the cell environment. Scale bar, 200 μm.

As an important property of microrobots for drug delivery, the movability of the microrobots has also been demonstrated in this work. We first tested the swimming performance of the microrobots within a rotating magnetic field of 20 mT. By gradually increasing the frequency of the applied rotating magnetic field from 1 to 10 Hz, it was demonstrated that the movement speed of the microrobot increased first and then decreased, and the speed reached the maximum at the frequency of 6 Hz. Furthermore, the targeting capability of the microrobots was also demonstrated within a microchannel and cell environment. Figure 7A shows the motion of an ABF-MOF(FA)-DOX microrobot in a cross-microchannel. Under a rotating magnetic field with the intensity of 15 mT and the rotation frequency of 3 Hz, the microrobot can smoothly swim in the microchannel, and the moving direction can be changed by adjusting the direction of the magnetic field rotation axis, so as to realize the timely lane-changing swimming in the microchannel. Moreover, the microrobot has also been manipulated in a cancer cells environment. As depicted in Fig. 7B, in a petri dish filled with MCF-7 cells, the motion of a microrobot was demonstrated within a smooth, narrow path that was reserved in advance. With the control of a rotating magnetic field (20 mT, 2 Hz), the microrobot can move flexibly toward a corner that is surrounded by cells and difficult to reach. The corresponding movies are provided in the Supplementary Materials (Movies S1 and S2). These results indicate that our microrobots can be accurately manipulated by a magnetic field and perform specific tasks.

### Application demonstration

**Fig. 8.**
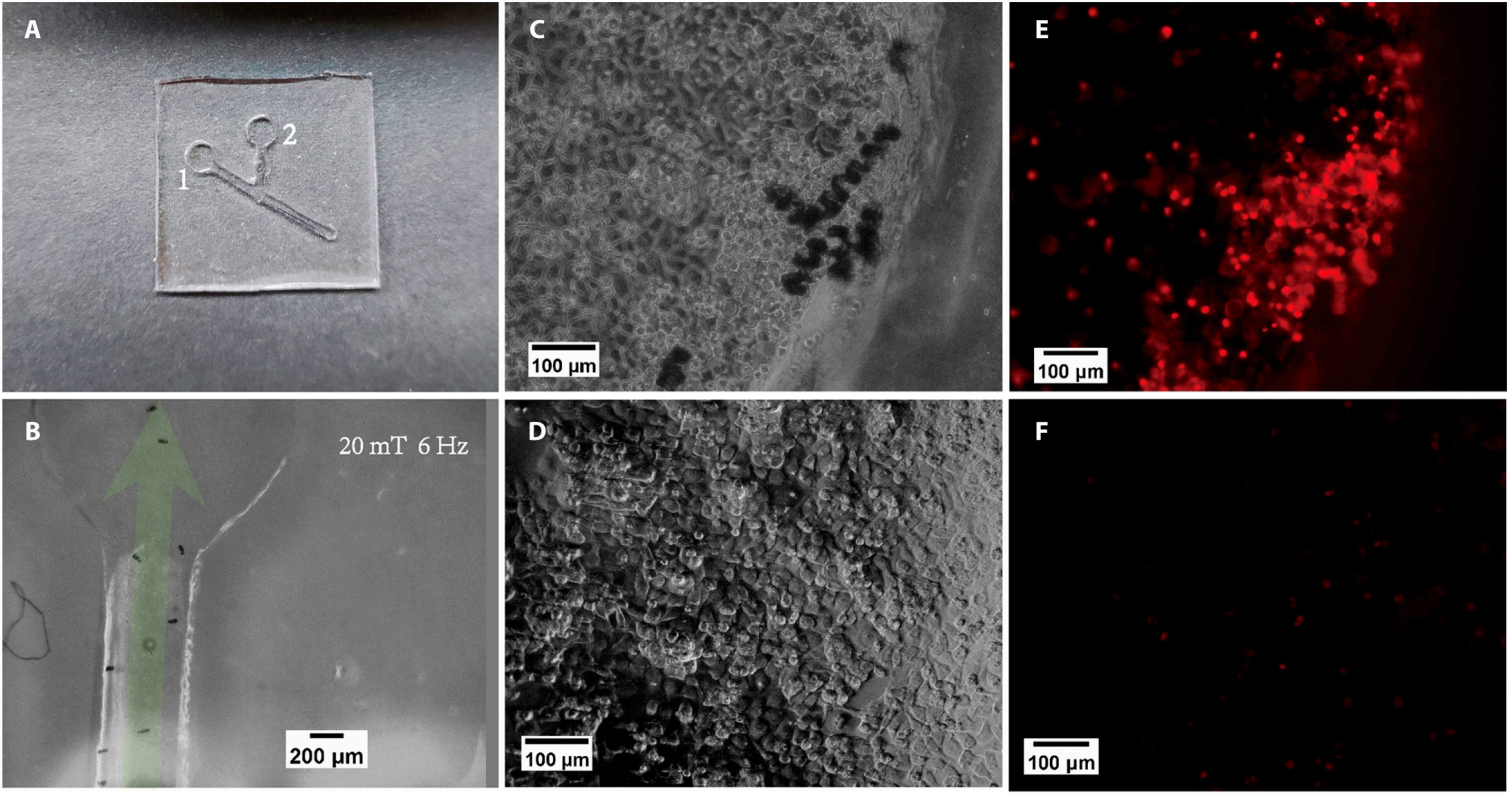
(A) A photo of the Y-shape microchannel. (B) A snapshot of microrobots being navigated in the Y-shape microchannel. (C and D) Bright-light images of hole 1 and hole 2 after the entire 48-h incubation, respectively. (E and F) Fluorescence images of dead cells in hole 1 and hole 2 after the entire 48-h incubation, respectively.

To demonstrate the anticancer effect of the folate-modified microrobots right after they have been navigated to a target location with a magnetic field, an application demonstration experiment was conducted in a Y-shaped PDMS microchannel (Fig. 8A). As shown in Fig. 8B and Movie S3, the magnetic navigation of a group of microrobots toward hole 1 within the microchannel was demonstrated. The survival states of the cancer cells after the entire 48-h incubation are indicated by both bright-light and fluorescence images (Fig. 8C to F). As cells would shrink and become round after death, many dead cells around the microrobots in hole 1 were observed (Fig. 8C), while the cells grew normally in hole 2, where no microrobots reached (Fig. 8D). Furtherly, the dead cell staining results also show that the density of dead cells around microrobots in hole 1 (Fig. 8E) is remarkably higher than the density of dead cells in hole 2 (Fig. 8F), where no microrobot exists. The results proved that, after being navigated to the designated location by a magnetic field, DOX-loaded folate targeting magnetic microrobots (ABF-MOF(FA)-DOX) can produce an obvious anticancer effect within 24 h.

## Conclusion

In conclusion, we proposed a magnetic microrobot platform with FA targeting function for enhanced cancer cell inhibition. These microrobots can transport drugs to cancer cell regions and increase the ingestion of drugs by cancer cells through receptor-ligand-mediated endocytosis. Due to the high drug loading capacity of GelMA hydrogel and MOF porous structures, drug and FA were successfully loaded in the hydrogel network and MOF nanoparticles of the microrobots, respectively. The successful loadings have been confirmed by UV/visible spectroscopy and microscopic images. The MTT assay and the live/dead staining experiments demonstrated that the existence of FA can substantially enhance the inhibition ability of therapeutic agents to cancer cells activity. Besides, the live/dead staining experiments also confirmed the good biocompatibility of the folate-modified microrobots when no DOX was loaded inside. The motion experiment proved that the microrobots can achieve directional movement in a cross-microchannel and cell environment under the actuation of a rotating magnetic field. In addition, an application experiment in the Y-shaped channel successfully demonstrated both the magnetic actuation and the enhanced targeted anticancer effect of folate-modified magnetic microrobots entirely. With the advantages of high loading capacity, controllable navigation, and enhancing cancer cell targeting and inhibition, the proposed folate-targeting magnetic microrobot system hold great potential in clinical cancer treatment.

## Data Availability

The authors confirm that the data supporting the findings of this study are available within the article and its supplementary materials.

## Supplementary Materials

Supplementary Materials Data file: Supporting Information. Audio files: Movies S1 to S3.
