# Supplementary material for: Magnetic Microrobots with Folate Targeting for Drug Delivery
Source: Cyborg Bionic Syst. 2023 May 5;4:0019. doi: 10.34133/cbsystems.0019 (PMC10202387; doi:10.34133/cbsystems.0019)
Supplement: Supplementary Materials — Data file: Supporting Information. Audio files: Movies S1 to S3. [file cbsystems.0019.f1.zip › Revised SI_CleanVersion.docx]

**Supporting information**

**Magnetic Microrobots with Folate Targeting for Drug Delivery**

Min Ye,^1^ Yan Zhou,^1^ Hongyu Zhao,^1^ Xiaopu Wang^1^*

*^1^Shenzhen Institute of Artificial Intelligence and Robotics for Society (AIRS), The Chinese University of Hong Kong, Shenzhen, Guangdong 518129, China*

Correspondence should be addressed to Xiaopu Wang; [wangxiaopu@cuhk.edu.cn](mailto:wangxiaopu@cuhk.edu.cn)

**Experiment section**

**Synthesis of gelatin methacryloyl (GelMA):**

**

**

The synthesis of GelMA was performed as described by Van Den Bulcke et al^1^. Type-B gelatin was dissolved in the PBS buffer solution at 45 ℃, then the MMA was added subsequently. Here, the ratio of gelatin and MMA was 1:10. After reacting for 12 h, the reaction solution mixture was dialyzed in NaHCO_3_ (0.2 M) for 24 h and in DI water for another 24 h at 45 ℃ with 4 times of water change per day. Finally, a white solid was obtained after lyophilization with [lyophilizer](javascript:;) (scientz-12N/A). NMR test validated that the substitution degree of the obtained GelMA is 90 %.

**Synthesis of Sodium 3,39-((((1E,19E)-(2-oxocyclopentane-1,3-diylidene) bis**

**(methanylylidene))bis(4,1-phenylene))bis(methylazanediyl))**

**dipropanoate (P2CK):**


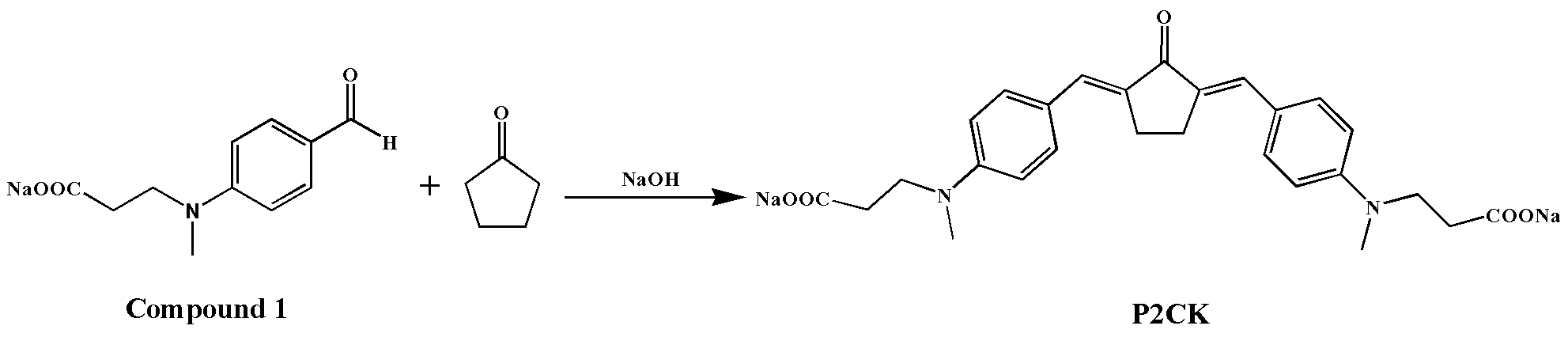


The two-photon photoinitiator (P2CK) was synthesized according to the protocol reported by the Liska group^2^. Briely, 3-((4-formylphenyl)-(methyl)-amino) propanenitrile (47.8 mmol) and sodium hydroxide (140 mmol) were dissolved in 200 mL DI water. After reflowed for 5 h, the reaction solution was cooled and filtrated. A [diluted hydrochloric acid](javascript:;) (HCl) was dropped into the filtrate slowly while being stirred, until there was no precipitate formed. Then the precipitate was washed with DI water for several times. After drying, a yellow compound 1 was obtained.

Compound 1 (4.25 mmol), sodium hydroxide (0.25 mmol) and benzaldehyde (2.26 mmol) were dissolved in 10 mL DI water and refluxed for 4 h at 80 ℃. After the reaction solution was cooled to room temperature, 20 mL of [absolute ethyl alcohol](javascript:;) and 1M HCl was added until there was no more precipitate formed. Then the red precipitate was obtained after being filtered and dried in vacuum. 20 mL DI water was added to the red precipitate and then 0.1 M NaOH was slowly dropped inside until the pH reached 8.5. Here, the product was dissolved in water, and the undissolved solids were filtered off. The target P2CK would be obtained by lyophilizating the filtrate.The chemical structure of the obtained P2CK was validated by NMR test. MP: ^1^H NMR (500 MHz, D2O) δ =7.10 (d, J = 8.5 Hz, 4H), 6.92 (s, 1H), 6.37 (d, J = 8.6 Hz, 2H), 3.29 (t, J=7.8Hz, 2H), 2.52 (s, 3H), 2.35 (s, 2H), 2.19(t, J=7.7 Hz, 2H).

**References**

1. Van Den Bulcke, A. I.; Bogdanov, B.; De Rooze, N.; Schacht, E. H.; Cornelissen, M.; Berghmans, H. J. B., Structural and rheological properties of methacrylamide modified gelatin hydrogels. **2000,** *1* (1), 31-38.

2. Li, Z.; Torgersen, J.; Ajami, A.; Mühleder, S.; Qin, X.; Husinsky, W.; Holnthoner, W.; Ovsianikov, A.; Stampfl, J.; Liska, R. J. R. a., Initiation efficiency and cytotoxicity of novel water-soluble two-photon photoinitiators for direct 3D microfabrication of hydrogels. **2013,** *3* (36), 15939-15946.
