# Supplementary material for: Magnetic Microrobots with Folate Targeting for Drug Delivery
Source: Cyborg Bionic Syst. 2023 May 5;4:0019. doi: 10.34133/cbsystems.0019 (PMC10202387; doi:10.34133/cbsystems.0019)
Supplement: Supplementary Materials — Data file: Supporting Information. Audio files: Movies S1 to S3. [file cbsystems.0019.f1.zip › Supporting Information.docx]

**Supporting information**

**Microrobot with Magnetic Actuation and Folate Targeting for Drug Delivery**

Min Ye,^1^ Yan Zhou,^1^ Hongyu Zhao,^1^ Xiaopu Wang^1^*

*^1^Shenzhen Institute of Artificial Intelligence and Robotics for Society (AIRS), The Chinese University of Hong Kong, Shenzhen, Guangdong 518129, China*

Correspondence should be addressed to Xiaopu Wang; [wangxiaopu@cuhk.edu.cn](mailto:wangxiaopu@cuhk.edu.cn)

**Experiment section**

**Synthesis of gelatin methacryloyl (GelMA):**

**

**

Type-B gelatin was dissolved in the PBS buffer solution at 45 ℃, then the MMA was added subsequently. Here, the ratio of gelatin and MMA was 1:10. After reacting for 12 h, the reaction solution mixture was dialyzed in NaHCO_3_ (0.2 M) for 24 h and in DI water for another 24 h at 45 ℃ with 4 times of water change per day. Finally, a white solid was obtained after lyophilization with [lyophilizer](javascript:;) (scientz-12N/A). NMR test validated that the substitution degree of the obtained GelMA is 90% .

**Synthesis of Sodium 3,39-((((1E,19E)-(2-oxocyclopentane-1,3-diylidene) bis**

**(methanylylidene))bis(4,1-phenylene))bis(methylazanediyl))**

**dipropanoate (P2CK):**

**
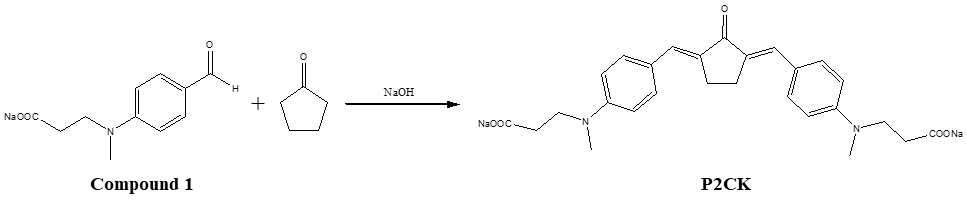
**

3-((4-formylphenyl)-(methyl)-amino) propanenitrile (47.8 mmol) and sodium hydroxide (140 mmol) were dissolved in 200 mL DI water. After reflowed for 5 h, the reaction solution was cooled and filtrated. A [diluted hydrochloric acid](javascript:;) (HCl) was dropped into the filtrate slowly while being stirred, until there was no precipitate formed. Then the precipitate was washed with DI water for several times. After drying, a yellow compound 1 was obtained.

Compound 1 (4.25 mmol), sodium hydroxide (0.25 mmol) and benzaldehyde (2.26 mmol) were dissolved in 10 mL DI water and refluxed for 4h at 80 ℃. After the reaction solution was cooled to room temperature, 20 mL of [absolute ethyl alcohol](javascript:;) and 1M HCl was added until there was no more precipitate formed. Then the red precipitate was obtained after being filtered and dried in vacuum. 20 mL DI water was added to the red precipitate and then 0.1 M NaOH was slowly dropped inside until the pH reached 8.5. Here, the product was dissolved in water, and the undissolved solids were filtered off. The target P2CK would be obtained by lyophilizing the filtrate. The chemical structure of the obtained P2CK was validated by NMR test. MP: ^1^H NMR (500 MHz, D2O) δ =7.10 (d, J = 8.5 Hz, 4H), 6.92 (s, 1H), 6.37 (d, J = 8.6 Hz, 2H), 3.29 (t, J=7.8Hz, 2H), 2.52 (s, 3H), 2.35 (s, 2H), 2.19(t, J=7.7 Hz, 2H).

**The characterization of MOF, MOF(FA) and ABF-MOF(FA)-DOX**


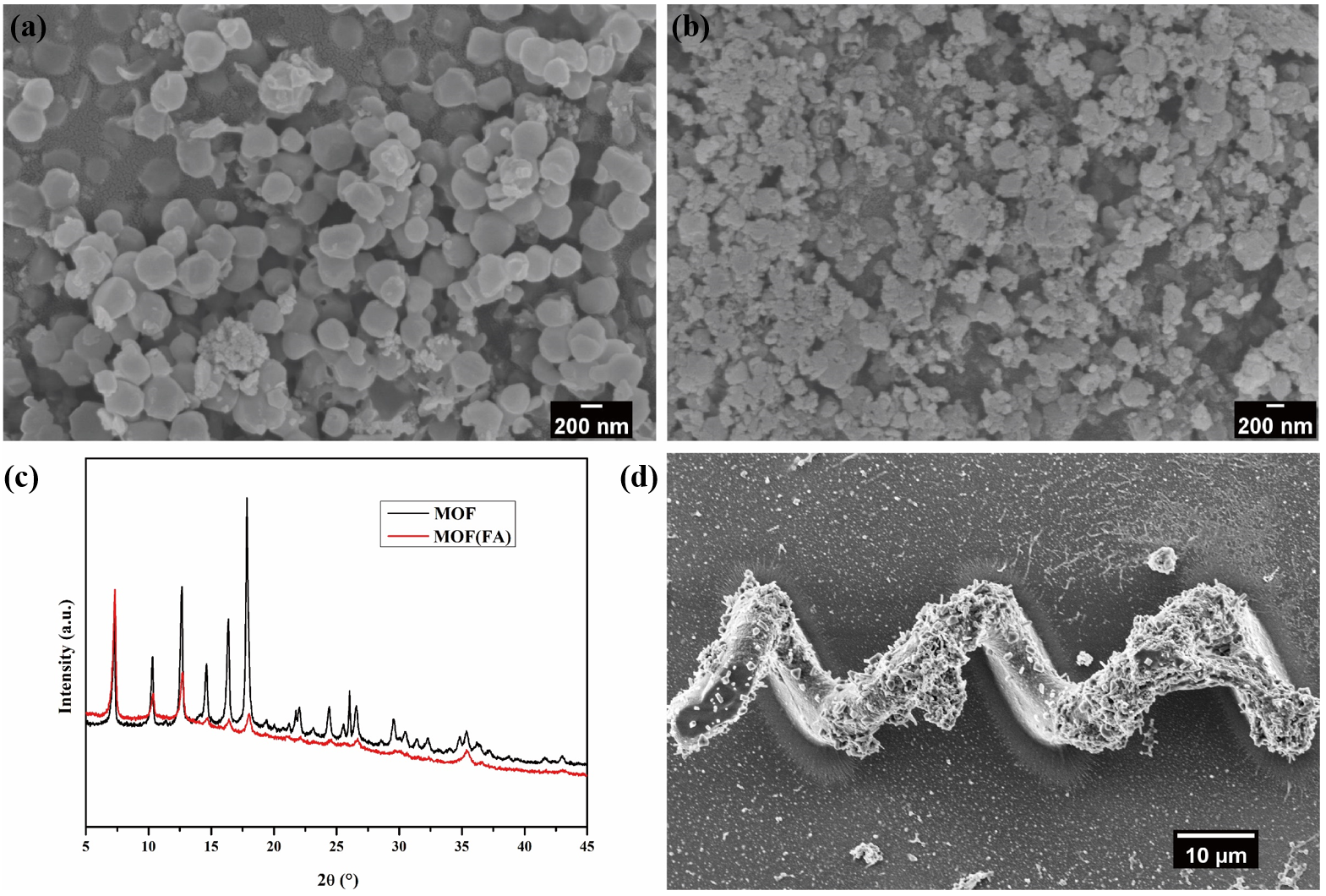


Figure S1. (a-b) the SEM images of MOF and MOF(FA), (c) XRD analysis of MOF and MOF(FA); (d) a SEM image of ABF-MOF(FA)-DOX

The crystalline structure of the MOF (Fe@ZIF-8) and MOF(FA) was analyzed using X-ray diffraction (XRD). As we can see in Figure S1(c), the intensity of some characteristic peaks of MOF would decrease when FA is loaded. This also corresponds to the result of the SEM image Figure S1(a-b), which descript the morphology change of MOF before and after FA loading. These changes might because of the low crystallinity of the MOF caused by the loading of drugs/cargos on the platforms^1^. The SEM image of ABF-MOF(FA)-DOX microrobot shows in Figure S1(d) proves the successful loading of MOF(FA) nanoparticles on the surface of GelMA based ABF structures.

**References**

1. Zeng, X.; Chen, B.; Song, Y.; Lin, X.; Zhou, S. F.; Zhan, G., Fabrication of Versatile Hollow Metal-Organic Framework Nanoplatforms for Folate-Targeted and Combined Cancer Imaging and Therapy. *ACS Appl Bio Mater* **2021,** *4* (8), 6417-6429.
